# Micro-computed tomographic assessment of the variability and morphological features of root canal system and their ramifications

**DOI:** 10.1590/1678-7757-2019-0393

**Published:** 2020-01-31

**Authors:** Jardel Francisco MAZZI-CHAVES, Yara Terezinha Corrêa SILVA-SOUSA, Graziela Bianchi LEONI, Alice Corrêa SILVA-SOUSA, Lucas ESTRELA, Carlos ESTRELA, Reinhilde JACOBS, Manoel Damião de SOUSA-NETO

**Affiliations:** 1 Universidade de São Paulo Faculdade de Odontologia de Ribeirão Preto Departamento de Odontologia Restauradora Ribeirão PretoSão Paulo Brasil Universidade de São Paulo, Faculdade de Odontologia de Ribeirão Preto, Departamento de Odontologia Restauradora, Ribeirão Preto, São Paulo, Brasil.; 2 University of Leuven Faculty of Medicine Departament of Imaging and Pathology Leuven Belgium University of Leuven, Faculty of Medicine, Departament of Imaging and Pathology, OMFS IMPATH Research Group, Leuven, Belgium.; 3 Universidade de Ribeirão Preto Faculdade de Odontologia Ribeirão PretoSão Paulo Brasil Universidade de Ribeirão Preto, Faculdade de Odontologia, Ribeirão Preto, São Paulo, Brasil.; 4 Universidade Federal de Goiás Faculdade de Odontologia GoiâniaGoiás Brasil Universidade Federal de Goiás, Faculdade de Odontologia, Goiânia, Goiás, Brasil.

**Keywords:** Dental pulp cavity, Microcomputed tomography, Anatomy, Tooth root, Endodontics

## Abstract

**Objectives:**

This study assessed the incidence and variability features of root canals system (RCS) and their ramifications according to Pucci & Reig (PR) (1944) and the American Association of Endodontists (AAE) (2017) by micro-computed tomography (μCT).

**Methodology:**

500 representative extracted human teeth of each tooth group (n=50) (maxillary/mandibular central and lateral incisors, canines, first and second premolars and molars) were scanned by μCT with a resolution of 26.70 μm. The reconstructed cross-sections images and the visualization of the continuous slices in the transversal axis were performed using DataViewer software. RCS were classified according to Pucci & Reig (main canal, collateral canal, lateral canal, secondary canal, accessory canal, intercanal, recurrent canal) and AAE (main canal, accessory canal, lateral canal). The apical deltas were assessed for both classifications. The prevalence of apical deltas was evaluated using the Chi-squared test (p<0.05).

**Results:**

According to PR, a higher incidence of lateral canals was observed in maxillary canines (10%), central incisors (8%) and first premolars (6%). Using AAE, the highest incidence of lateral canals was observed in the mandibular first premolars (85%), first and second molars (84%), lateral incisors (67%), canines (59%), and in maxillary first premolars (52%). Regarding accessory canals, the PR showed a frequency in 2% of the maxillary lateral incisors and maxillary and mandibular first premolars and 3% of mandibular first and second molars. On the other hand, the AAE showed the highest incidence of accessory canals in 86% of the maxillary first premolars, 71% in mandibular lateral incisors, 69% in mandibular first premolars, 65% in mandibular canines, and 56% in maxillary canines. The PR showed the lowest incidence of apical deltas for all dental groups when compared with AAE (p=0.004). Interestingly, distal canals in maxillary molars showed a significant discrepancy between classifications (p=0.027).

**Conclusions:**

μCT enabled accurately describing the RC system and related ramifications, adding to the PR and AAE classifications, with some discrepancies reported for maxillary molars. Clinical Relevance This μCT study enabled a thorough description of the variability among root canals and their ramifications, including clinically relevant details on the presence and location of lateral canals and accessories in all human tooth groups, beyond the currently existing classification systems.

## Introduction

The success of endodontic treatment directly depends on complete cleaning, disinfection, shaping and three-dimensional (3D) filling of the root canal system (RCS).^1 - 8^ The subsequent steps should be meticulously performed to ensure the total removal of healthy or necrotic tissue and to eliminate microorganisms and related subproducts from root canals.^7 - 11^ Thus, a deepened knowledge of the 3D RCS morphology contributes to the diagnosis and establishment of the endodontic therapy protocol, supporting the prognosis and obtaining a successful treatment.^4 , 5 , 8 , 12 - 17^

Studies on the morphology of root canals of human permanent teeth^1 , 13 , 18 , 19^ have shown that the number and classification of root canals could vary in each teeth group, according to ethnicity, sex and between populations, within the same population as well as individually, in each person.^4 , 5 , 15 , 16 , 20 - 23^

The accessory (extra or additional) root canals refers to an anatomical development variation, with the presence of additional root canals compared to the original dental anatomy previously described in the literature.^4^ This anatomical variation is very common in human teeth (primary and permanent) and interconnects the main root canal or pulp chamber to the external root surface due to the trapping of periodontal blood vessels during the Hertwig root epithelial sheath formation and development.^24^ Thus, it is essential to know the internal dental anatomy and its variations for endodontic treatment, since the literature has reported the incidence of accessory canals between 40% and 80% in different dental groups.^24^

Therefore, to understand the complexity of the RCS, several studies have been conducted and numerous classifications have been proposed.^1 , 4 , 5 , 11 , 12 , 14 , 23 - 28^ However, it is noteworthy that many of these classifications are inconsistent, based on specific and/or reduced samples, with destructive methods enabling only two-dimensional analysis, hampering the standardization and its use.^5 , 6 , 14 - 16^ Thus, most anatomical studies have been based on two traditional classifications: Pucci & Reig^25^ (1944) and the American Association of Endodontists (AAE)^28^ (2017), which include in a clear, objective and didactic way the description of the types of root canals in all human tooth groups.^12 , 25 , 28^ The definition proposed by PR is more complete and classifies the root canal system in main canal, collateral canal, lateral canal, secondary canal, accessory canal, intercanal, recurrent canal and apical deltas.^25^ On the other hand, the AAE classification is simpler and more objective, classifying the anatomical variations of the root canals in main canal, accessory canal, lateral canal and apical deltas.^28^

In order to enable visualization and characterization of RCS, micro-computed tomography (μCT) has become a reference standard enabling a fast and detailed, noninvasive, nondestructive, and high precision three-dimensional analysis of the RCS, ensuring the consistency of the results obtained.^4 , 5 , 8 , 11 , 14 , 17 , 23 , 29 - 31^

Many studies have used μCT to describe the RCS classification in several groups of teeth in upper and lower jaw in different populations. Until now, none of these reports have addressed this variability considering an in depth three-dimensional characterization of the RCS between different classifications. Thus, this study aims to provide high-resolution 3D analysis of the morphological features of root canal systems and their ramifications. This highly detailed morphological assessment with micro-computed tomography and advanced software analysis was used to add a refinement to both classical classification systems: Pucci & Reig^25^ (1944) and American Association of Endodontists^28^ (2017) in human permanent dentition.

## Methodology

### Sample Selection

After the approval by the Research Ethics Committee (CAAE n.º 0072.0.138.000-09) for the use of 500 human teeth extracted with reasons unrelated to this study from a representative Brazilian subjects, 50 subsets were selected for each tooth group (n=50) (maxillary and mandibular: central and lateral incisors, canines, first and second premolars, and molars), with complete rhizogenesis, root structure completely formed, and without caries or previous endodontic treatment. All teeth were stored in a 0.1% thymol solution for 24 hours. Afterwards, teeth were washed in running water for 24 hours and their external root surface were cleaned by ultrasonic scaling (Dabi Atlante Ltda., Ribeirão Preto, SP, Brazil). After this procedure, the teeth were kept in 1% sodium hypochlorite solution for 2 hours to remove the remaining pulp tissues to avoid interference in the images processing during the micro-computed tomography (μCT) scanning.

### Micro-CT Analysis

Thus, to acquire μCT images, teeth were placed in an individually customized apparatus and the scanning was made separately in a SkyScan 1174 μCT device (Bruker-microCT, Kontich, Belgium) with isotropic voxel size of 26.70 μm. The scanning parameters used were 50 kV, 800 μA, 180° rotation around the vertical axis, rotation step of 1°, and a 0.5 mm-thick aluminum filter, rendering a scan time of 25 minutes, approximately. The three-dimensional reconstruction of the cross-sections from the angular projection images was performed by NRecon v.1.7.1.0 software (Bruker-microCT, Kontich, Belgium). The applying parameters included ring artifact reduction of 5, beam hardening correction of 40%, smoothing of 3, and an attenuation coefficient between 0.001 and 0.15.

Root canal analysis was performed by DataViewer v.1.5.4.0 software (Bruker-microCT, Kontich, Belgium), through visualization of continuous slices in the transversal axis of the specimens. Furthermore, using the CTAn v.1.17.7.2+ software (Bruker microCT, Kontich, Belgium), 3D models were generated from the specimens in the P3G format and, for qualitative analysis, a realistic view of the root canals was created using 3D models of the roots and root canals. All analyses were performed by the same observer.

### Classification of the root canals and their ramifications

The classification of root canals and their ramifications was performed considering the proposal of Pucci & Reig^25^ (1944) and the American Association of Endodontists^28^ (2017). The root canals were classified following the terminology proposed by PR: *main canal:* canal that normally passes through the dental axis and can reach without interruption the root apex; *collateral canal* : it follows a path parallel to the main canal, being able to independently reach the root apex; *lateral canal* : it connects the main canal to the external root surface; *secondary canal* : it leaves the main canal in its apical portion and ends in the periapical region of the tooth; *accessory canal* : it derives from a secondary canal and follows to the external root surface; *intercanal* : it connects the main canals, is located in the dentin, and does not reach the cement area; *recurrent canal* : it leaves the main canal and returns to it, the path is exclusive to dentin; *reticular canal* : it is the interlacing of three or more canals that run almost parallel, by branches of the intercanal, with cross-linked aspect and; *apical deltas* : multiple derivations that are close to the same root apex and that leave the main canal to end in the apical zone.

On the other hand, the AAE proposed a simpler and more objective classification. Thus, the canals could be classified in: *main canal* : it is a passage or course in the tooth root extending from the pulp chamber to the apical foramen; may be narrow, has lateral branches and/or exhibit irregular morphology; *accessory canal* : any branch of the main pulp canal or chamber that communicates with the external surface of the root; *lateral canal* : it is an accessory canal located in the coronal or middle third of the root, usually extending horizontally from the main canal space and; *apical deltas* : it is a pulp canal morphology in which the main canal divides into multiples accessory canals at or near the apex.

### Statistical analysis

The data were examined for normal distribution (Shapiro–Wilk test, p>0.05) and homogeneity of variance (Levene’s test, p>0.05). The prevalence of apical delta was assessed using the Chi-squared test (p<0.05), using the Sigmaplot 11.0 software package (Systat Software Inc., San Jose, CA, USA), with the significance level set at α=0.05.

## Results

### Pucci & Reig classification of root canals

**Figure 1:**
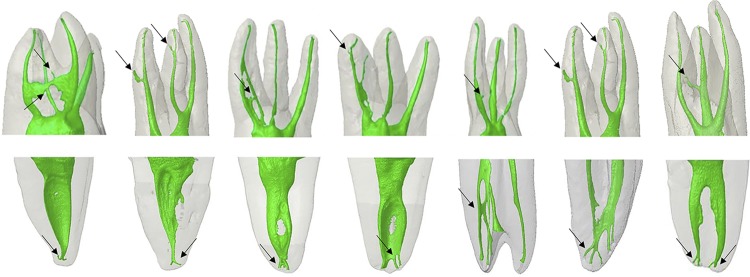
3D models of maxillary and mandibular teeth showing the morphology of the root canal system according to the root thirds. The presence of main, accessories, lateral, secondary, collateral, intercanal, recurrent and reticular canals, and apical deltas was indicated by black arrows

**Figure 2:**
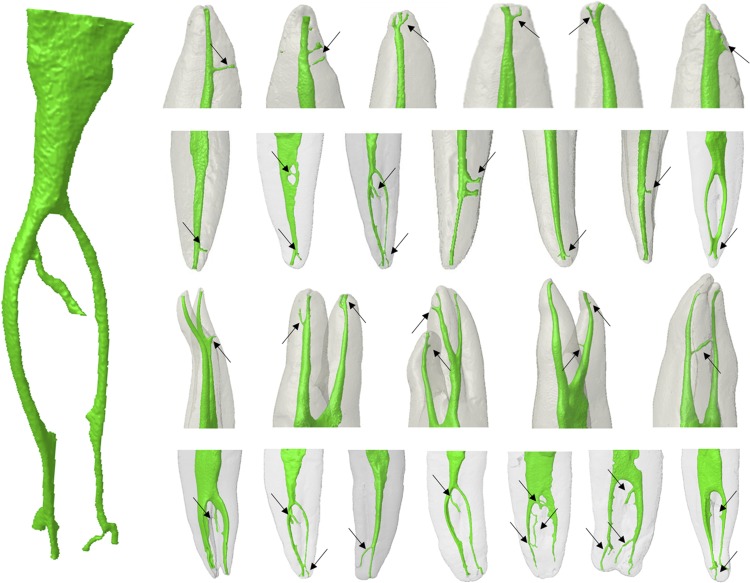
3D models of maxillary and mandibular teeth showing the morphology of the root canal system according to the root thirds. The presence of main, accessories, lateral, secondary, collateral, intercanal, recurrent and reticular canals, and apical deltas was indicated by black arrows

**Figure 3:**
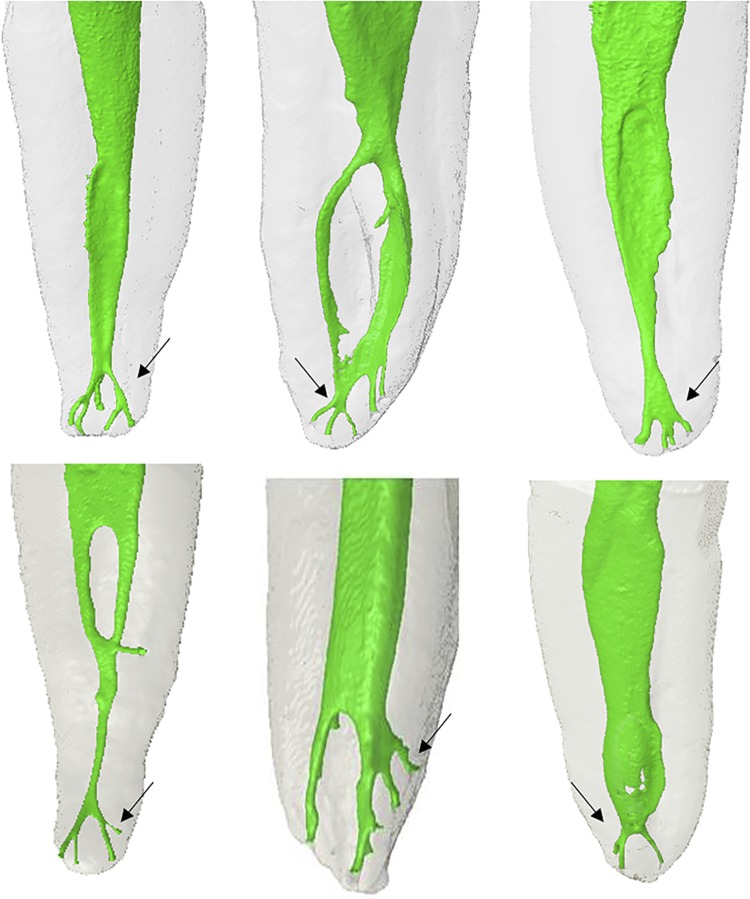
3D models of human teeth showing the variability of the root canal system morphology. The black arrows indicate the variability of apical deltas

The percentage of root canals types found according to the PR classification are shown in Table 1 . The assessment evidenced the presence of a main canal in 100% of the assessed dental groups, except for the second mesiobuccal canal in the maxillary first and second molars, which showed an incidence of 87% and 75%, respectively. There was a higher incidence of lateral canals in the maxillary canines (10%), followed by maxillary central incisors (8%) and maxillary first premolars (6%). The presence of secondary canals was 46% in the maxillary canines, 22% in the maxillary lateral incisors, 14% in the maxillary central incisors, 3% in the mandibular first premolars, and between 8% and 10% in the mesiobuccal root of the mandibular first and second molars. It was possible to observe the presence of collateral canals in 2% of the maxillary central incisors, in 1% of the mandibular central incisors and in 3% of the mandibular lateral incisors. The accessory canals were observed in 2% of the maxillary lateral incisors and maxillary and mandibular first and second premolars and between 3% and 4% of mandibular first and second molars. The highest incidence of apical deltas was found in 6% of the maxillary canines, in 4% of maxillary second premolars, in 6% of the mesiobuccal root of maxillary first molars and in 7% of the mandibular first premolars. In addition, recurrent canals were found in 2% of the maxillary lateral incisors and intercanals in 2% of the maxillary first molars and mandibular canines ( Table 1 , Figures 1–3 ).

**Table 1:** Percentage distribution of root canal types found according to the Pucci & Reig (1944) classification

Dental group		Root canal	Main	Colateral	Lateral	Secondary	Accessory	Recurrent	Intercanal	Apical delta
Maxillary	CI		100%	2%	8%	14%	0%	0%	0%	2%
	LI		100%	0%	4%	22%	0%	2%	0%	4%
	C		100%	0%	10%	46%	0%	0%	0%	6%
	1^st^ PM	B	100%	0%	4%	5%	2%	0%	0%	2%
		P	100%	0%	6%	2%	0%	0%	0%	2%
		Single	100%	0%	2%	4%	0%	0%	0%	4%
	2^nd^ PM	B	100%	0%	2%	3%	0%	0%	0%	3%
		P	100%	0%	0%	0%	0%	0%	0%	2%
		B	100%	0%	0%	2%	0%	0%	2%	6%
	1^st^ M	MB	87%	0%	0%	0%	0%	0%	2%	2%
		D	100%	0%	0%	0%	0%	0%	0%	0%
		P	100%	0%	0%	0%	0%	0%	0%	0%
		B	100%	0%	0%	3%	0%	0%	0%	4%
	2^nd^ M	MB	75%	0%	0%	0%	0%	0%	0%	2%
		D	100%	0%	0%	0%	0%	0%	0%	0%
		P	100%	0%	0%	0%	0%	0%	0%	0%
Mandibular	CI		100%	1%	3%	2%	0%	0%	0%	2%
	LI		100%	3%	2%	3%	2%	0%	0%	0%
	C		100%	0%	1.8%	0%	0%	0%	2%	0%
	1^st^ PM	Single	100%	0%	4%	3%	2%	0%	0%	7%
	2^nd^ PM	Single	100%	0%	3%	2%	2%	0%	0%	5%
	1^st^ M	MB	100%	0%	2%	10%	3%	0%	0%	4%
		ML	100%	0%	2%	4%	2%	0%	0%	4%*
		D	100%	0%	4%	6%	2%	0%	0%	4%
	2^nd^ M	MB	100%	0%	2%	8%	2%	0%	0%	2%
		ML	100%	0%	3%	4%	2%	0%	0%	3%
		D	100%	0%	5%	4%	3%	0%	0%	4%
		Single	100%	0%	0%	0%	4%	0%	0%	3%

CI: central incisors; LI: lateral incisors; C: canines; PM: premolars; M: molars; B: buccal; P: palatal; MB: mesiobuccal; ML: mesiolingual; D: distal; Bold numbers: statistical difference between classifications; *difference between canals in both classification

### American Association of Endodontists classification of root canals

Table 2 shows the percentage of types of root canals found according to the AAE. Regarding the main canal, the AAE classification showed the same results observed in the PR classification. The accessory canal showed the highest incidence in 86% of palatal roots of maxillary first premolars, followed by 75% of incidence in single maxillary second premolars, 71% in mandibular lateral incisors, 69% in single mandibular first premolars, 65% in the mandibular canines, 62% in single mandibular second premolars and 56% in maxillary canines.

**Table 2:** Percentage distribution of root canal types found according to the AAE (2017) classification

Dental group		Root canal	Main	Accessory	Lateral	Apical delta
	CI			24%	8%	2%
	LI			28%	4%	4%
	C			56%	10%	6%
	1^st^	B	100%	60%	45%	3%
	PM	P	100%	86%	52%	3%
	2^nd^	Single	100%	75%	48%	4%
Maxillary	PM	B	100%	50%	35%	3%
		P	100%	44%	47%	2%
	1^st^	B	100%	34%	44%	4%
	M	MB	87%	25%	15%	0%
		D	100%	46%	38%	12%*
		P	100%	10%	9%	5%
	2^nd^	B	100%	36%	39%	2%
	M	MB	75%	22%	10%	0%
		D	100%	31%	33%	10%*
		P	100%	7%	5%	3%
Mandibular	CI		100%	54%	45%	9%
	LI		100%	71%	67%	4%
	C		100%	65%	59%	6%
	1^st^	Single	100%	69%	85%	7%
	PM					
	2^nd^	Single	100%	62%	78%	5%
	PM					
	1^st^	MB	100%	35%	56%	8%
	M	ML	100%	46%	84%	12%*
		D	100%	29%	47%	5%
	2^nd^	MB	100%	25%	45%	5%
	M	ML	100%	34%	76%	5%
		D	100%	31%	37%	4%
		Single	100%	45%	34%	7%

CI: central incisors; LI: lateral incisors; C: canines; PM: premolars; M: molars; B: buccal; P: palatal; MB: mesiobuccal; ML: mesiolingual; D: distal; Bold numbers: statistical difference between classifications; *difference between canals in both classification

On the other hand, there was a higher incidence of lateral canals in the mandibular teeth, as follows: first premolars (85%), second premolars (78%), mesiolingual roots of first and second molars (84% and 76%, respectively), lateral incisors (67%), canines (59%), and palatal roots of maxillary first premolars (52%). Apical deltas were found more frequently in 12%, 9%, 7% and 6% of the maxillary and mandibular first molars, mandibular central incisors, mandibular first premolars and maxillary and mandibular canines, respectively ( Table 2 , Figures 1-3 ).

The PR showed the lowest incidence of apical deltas for all dental groups when compared with AAE (p=0.004). When teeth group was assessed, only distal canals in maxillary first and second molars showed statistical difference between the two classification systems (p=0.027).

## Discussion

The complex anatomy of the RCS, including the presence of accessory, lateral, and apical deltas is directly related to the failure of the endodontic treatment, for these areas are difficult to access during the cleaning/shaping and disinfection of the root canals. Thus, for this difficult access, some organic and necrotic tissues remain inside the root canals, as well as a significant number of bacteria, which can proliferate continuously and rapidly affect the periapical region.^3 , 5 - 7 , 11 , 12 , 16 , 23 , 25 , 31 , 32^

Therefore, diagnosis, cleaning, and disinfection of these branches are extremely important and considered as the main objective of endodontic therapy, avoiding reinfection of the RCS, periradicular tissues and, endodontic-periodontic problems.^3 , 5 , 7 , 11 , 12 , 31 - 33^

In relation to the classification of canals, some studies classify the canals according to Pucci & Reig^25^ (1944) or according to the AAE^28^ (2017), and no consensus is reached in the literature regarding the standardization of classifications and terminologies, which may hamper the comparison between the different studies of anatomy. In the literature, few studies mention the type of classification used.^12 , 34 , 35^ Thus, this study performed both AAE^28^ and PR^25^ classifications, to compare the results obtained in the studies present in the literature.

The classification of the types of canals according to PR^25^ showed the presence of lateral canals in 1.8%-10% of the assessed teeth, and the higher incidence can be observed in the upper canines. Accessory canals were observed in 2%-3% with higher prevalence in the mandibular molars. These results can be observed in other studies in the literature, which found a similar prevalence.^12 , 14 , 31 , 32 , 36 , 37^ It was also possible to observe the presence of collateral canals (0%-3%), recurrent canals (0%-2%), intercanals (0%-2%) and secondary canals (2%-46%). Following the PR classification, De-Deus^12^ (1975), in his well-established study about root canals through diaphanization technique, observed lateral and accessory canals in 10.4% and 0.6% of the sample, respectively.

The highest percentage of anatomical apical variations found in this study may be attributed to more accurate assessment method, when compared with De-Deus^12^ (1975) study, which assessed the presence of these canals by diaphanization. Furthermore, this study was concerned in maintaining the teeth inside of 1% sodium hypochlorite solution to dissolve tissue in order to avoid interference in the analysis of the images.

On the other hand, the AAE^27^ classification evidenced an incidence of accessory canals between 10% and 86% and lateral canals in 4-85% of the assessed teeth, which can be found similarly in other studies.^9 , 11 , 32 , 34 , 35 , 38^ The presence of apical deltas ranged between 2% and 7% for the Pucci & Reig^25^ classification and between 2% and 12% for AAE^28^ , with a higher prevalence in the distobuccal root canals of maxillary molars. Although most studies do not mention the type of classification used, the data obtained in this study for apical deltas agree with what has been previously established in the literature.^1 , 6 , 8 , 12 , 14 , 18 , 31 , 34 , 38 - 40^

This study showed a high incidence of apical deltas in all dental groups assessed in the literature.^6 , 24^ These studies found a highest incidences in maxillary first and second premolars and molars when compared with the anterior teeth, corroborating the results obtained in this study.^1 , 6 , 24 , 38 - 40^ Also, it is noteworthy that the accessory canals were observed more frequently in the mesiobuccal roots of the maxillary molars (first and second) and in the mesial roots of the mandibular molars (first and second), confirming the previous results published in the literature.^1 , 6 , 24 , 38 - 40^

According to the classifications used in this study, the highest incidence of accessory canals was located in the apical third of the assessed teeth. These results are associated with the previous studies by Degerness & Bowles^39^ (2008) and Ordinola-Zapata, et al.^36^ (2019), who found the incidence of 80% and 76%, respectively, of accessory canals in the apical third of mesiobuccal roots in the maxillary molar.^36 , 39^

The increased incidence of lateral and accessory canals and apical deltas in the AAE^28^ (2017) classification compared with Pucci & Reig^25^ (1944), is due to the term referring to every branch of the main canal that connects with the outer root surface in this new classification, differing only the apical delta term that refers to a branch of main canal into multiples canals in the apical region.

Different studies describe a diversity of RCS. The latter reflects human variability and the acuity of different methodologies,^19^ as well as the use of different classifications. According to these data, the prevalence of lateral, secondary and apical delta canals is extremely high, regardless of the classification used. This finding has important clinical implication and should be considered during medical procedures.^1 , 4 - 6 , 8 , 9 , 11 , 12 , 14 , 15 , 18 , 23 , 32 - 35^ Furthermore, failure of endodontic treatment could be attributed to the persistent intraradicular infection in untreated canals, dentinal tubules, or complex RCS irregularities, such as lateral, secondary and apical deltas.^3 , 7 , 33^ This information is essential to optimize and to individualize the therapeutic approaches related to cleaning, instrumenting and filling RCS of various teeth groups.^4 - 9 , 11 , 16 , 23 , 31^

Although μCT provides high resolution and acuity 3D images due to the high energy parameters used (kVp and mA) and smaller voxel sizes, which result in long scanning times and high radiation doses, its use is limited to laboratory studies. However, these studies have supported the development of clinical research, and consequently the diagnosis of anatomical variations and the determination of endodontic treatment protocols.^2 , 8 , 10 , 14 , 17 , 24^

This μCT study enabled a thorough description of the variability among root canals and their ramifications, including clinically relevant details on the presence and location of lateral canals and accessories in all human tooth groups, beyond the current classification systems.

This detailed information may enable endodontists to accurately assess endodontic treatment needs and decide which tooth-specific treatment approach use. Surely when dealing with complex tooth morphologies, a 3D diagnostic approach might be necessary for tooth-specific pre-endodontic diagnostics and endodontic guidance. Although pieces of evidence exist and some clinical cone-beam computed tomography (CBCT) devices enable the visualization, more information is necessary to assess whether CBCT data are sufficient for the treatment. Nevertheless, such approach could optimize the endodontist clinical protocol for biomechanical preparation and filling of the RCS, ensuring a better outcome of the endodontic treatment.

Thus, according to the methodology used and the results obtained, diagnosis and planning of the endodontic treatment is expected to be conducted safely and effectively, ensuring more predictable results, based on the technological advances of the imaging methods of diagnostic — providing greater accuracy —, as well as the development of new devices and software for acquisition, reconstruction, and analysis of CBCT images.

## Conclusions

This study concluded that μCT enabled an accurate description of the variability of the RC system and related ramifications, supported by the PR and AAE classifications, with some discrepancies reported for upper molars.
